# Novel Gel Formulations as Topical Carriers for the Essential Oil of *Bidens tripartita* for the Treatment of Candidiasis

**DOI:** 10.3390/molecules23102517

**Published:** 2018-10-01

**Authors:** Monika Tomczykowa, Magdalena Wróblewska, Katarzyna Winnicka, Piotr Wieczorek, Piotr Majewski, Katarzyna Celińska-Janowicz, Róża Sawczuk, Wojciech Miltyk, Elżbieta Tryniszewska, Michał Tomczyk

**Affiliations:** 1 Department of Organic Chemistry, Faculty of Pharmacy with the Division of Laboratory Medicine, Medical University of Białystok, 15-230 Białystok, Poland; monika.tomczyk@umb.edu.pl; 2 Department of Pharmaceutical Technology, Faculty of Pharmacy with the Division of Laboratory Medicine, Medical University of Białystok, 15-222 Białystok, Poland; magdalena.wroblewska@umb.edu.pl (M.W.); kwin@umb.edu.pl (K.W.); 3 Department of Microbiological Diagnostics and Infectious Immunology, Faculty of Pharmacy with the Division of Laboratory Medicine, Medical University of Białystok, 15-269 Białystok, Poland; piowie@umb.edu.pl (P.W.); piotr.majewski@umb.edu.pl (P.M.); trynisze@umb.edu.pl (E.T.); 4 Department of Pharmaceutical Analysis, Faculty of Pharmacy with the Division of Laboratory Medicine, Medical University of Białystok, 15-222 Białystok, Poland; katarzyna.celinska-janowicz@umb.edu.pl (K.C.-J.); roza.sawczuk@umb.edu.pl (R.S.); wmiltyk@umb.edu.pl (W.M.); 5 Department of Pharmacognosy, Faculty of Pharmacy with the Division of Laboratory Medicine, Medical University of Białystok, 15-230 Białystok, Poland

**Keywords:** *Bidens tripartita*, essential oil, topical formulation, antifungal activity, genotoxicity

## Abstract

The genus *Bidens* L. (Asteraceae) refers to several species of plants used in traditional phytotherapeutic preparations. *B. tripartita*, also known as bur marigold, is the most familiar plant and has been known as a remedy for chronic dysentery. The hydrodistilled essential oil of the aerial parts of the Polish *B. tripartita* was analyzed using gas chromatography (GC) and gas chromatography-mass spectrometry (GC-MS) techniques. To exclude any potential toxic effects of the oil on human dermal fibroblasts, the MTT test (methyl thiazolyl tetrazolium) and COMET assay (single-cell gel electrophoresis) were performed. Novel gel formulations as topical carriers for essential oil obtained from *B. tripartita* were developed and characterized. The bioadhesive properties of the designed preparations in the ex vivo model using the skin of hairless mice were also evaluated. The therapeutic efficacy of the topical formulations is influenced by active phytoconstituents and vehicle characteristics. The antifungal properties of the essential oil of *B. tripartita* were also tested against *Candida* species, and this oil appears to be a promising topical anticandidal agent.

## 1. Introduction

A wide variety of plants found in Central and Eastern European flora have been identified through ethnopharmacological studies as antifungal agents. The genus *Bidens* L. (Asteraceae) consists of approximately 230 plant species that are used as anti-inflammatory, antiallergic, antidiabetic, analgesic and antioxidant agents [1,2,3,4]. The broad biological activity of *B. tripartita* is associated with phytochemicals, particularly, flavonoids, essential oils, and coumarins, as well as polyynes [5,6]. Gel formulations are very important group of local dosage forms and their use in therapy is becoming more widespread. They are characterized by many favourable features such as mucoadhesion, tixotropy, or ease of spreadability. Hydrogels constitute three-dimensional, hydrophilic polymer networks, whereas oleogels are composed of lipophilic fluid gelled with suitable gelling agents. Bigels combine the features of hydrogels and oleogels and they can be designed to deliver both lipophilic such as essential oils and hydrophilic drugs [7,8,9]. In this regard, the scope of this study was to develop and characterize different novel gel formulations containing the essential oil of *B. tripartita* herbs (BTEO). Additionally, the antifungal activity of the obtained formulations on many different *Candida* strains was examined. To exclude any potential toxic effects of BTEO on human dermal fibroblasts, MTT and COMET assays were performed for the first time.

## 2. Results and Discussion

The essential oil from dried herb of *Bidens tripartita* (BTEO) was obtained and analyzed by GC-MS using the pharmacopeia method. The amount of oil calculated for the BTEO was 0.19% (*v/w*). Ninety-eight compounds were identified in the oil of BTEO, which represents 94–98% of the total components detected. The oil was mainly composed of *p*-cymen (4.82%), β-linalool (2.85%), (Z)-cymen (2.60%) and α-fellandren (1.10%). The chemical composition of the essential oil depends on the many factors such as climate, method of extraction as well as vegetation stages. Kaskoniene et al. have studied the variation of essential oils composition of *B. tripartita* after supercritical CO_2_ extraction during three years 2009–2011. The obtained results revealed that the essential oils composition was highly dependent on the meteorological data such as temperature or precipitation. In contrast, the differences obtained in the essential oil composition are mainly due to a variety of extraction methods used [10,11].

**Figure 1:**
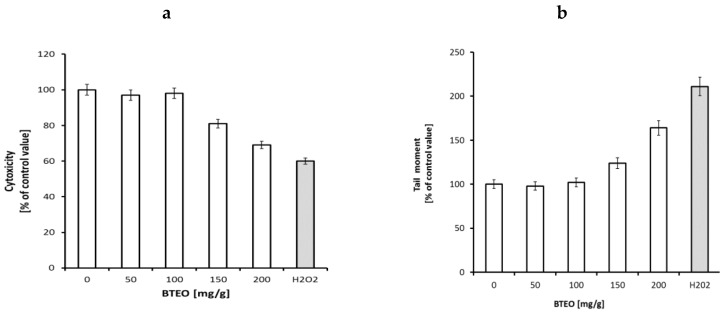
(**a**) Cytotoxicity of BTEO (50, 100, 150, 200 mg/g) in human dermal fibroblasts treated for 24 h. The MTT test is described in the Methods section. The mean of six independent experiments are presented; (**b**) COMET tail moments in human dermal fibroblasts treated with BTEO (50, 100, 150, 200 mg/g) for 24 h. The assay is described in the Methods section. H_2_O_2_ was used as a positive control.

To evaluate the potential toxicity of the oil, a MTT and COMET tests were conducted in human dermal fibroblasts culture. The MTT test indicated the dysfunction of mitochondria in cells treated with the tested compounds. The Comet assay is a fast and sensitive fluorescence method that allows the detection of DNA damage at the single cell level. The test provides a measure of DNA strand breaks. As a positive control, cells incubated with 100 μM of H_2_O_2_ were used. The results of both tests did not reveal significant changes in the cells treated for 24 h with 50 and 100 mg/g of BTEO (Figure 1a,b). Addition of the oil at concentrations of 150 and 200 mg/mL induced a significant increase of cytotoxic effect and tail moment in fibroblasts. This result suggests that BTEO is not toxic in human dermal fibroblasts culture at concentrations below 100 mg/mL. For further experiments, BTEO was used at a concentration of 100 mg/mL. The safety of the BTEO prompted us to prepare the formulations.

When designing local dosage forms, selection of the proper carrier is a crucial issue, as it affects characteristics of the final product. In this study, three gel formulations (a hydrogel, bigel and oleogel) containing BTEO were designed, and their physicochemical features were investigated. The designed formulations containing BTEO possessed homogenous consistency and no phase separation was observed. Essential oil tinged the formulations for yellow. In the case of the alginate hydrogel and the bigel, BTEO was uniformly suspended in the vehicle, while in the oleogel it was completely dissolved. The pH values of the obtained preparations with BTEO were in the range of 6.4 to 7.2 (Table 1), and no significant differences compared with the pH of the placebo formulations were noted. Moreover, the pH values of all of the formulations were suitable for topical delivery, enabling safe application to the skin without the risk of its irritation.

The viscosity and rheological properties of the dosage forms for topical administration are related to the spreading, application behavior and contact time with the skin surface. The obtained gel formulations containing BTEO were found to have different viscosity values (Table 1). The highest viscosity was noticed in the case of the bigel (11,833, mPa·s), and the alginate hydrogel (6879, mPa·s) possessed the lowest viscosity. The addition of essential oil increased viscosity values, which was particularly noticeable in the bigel formulation.

**Figure 2:**
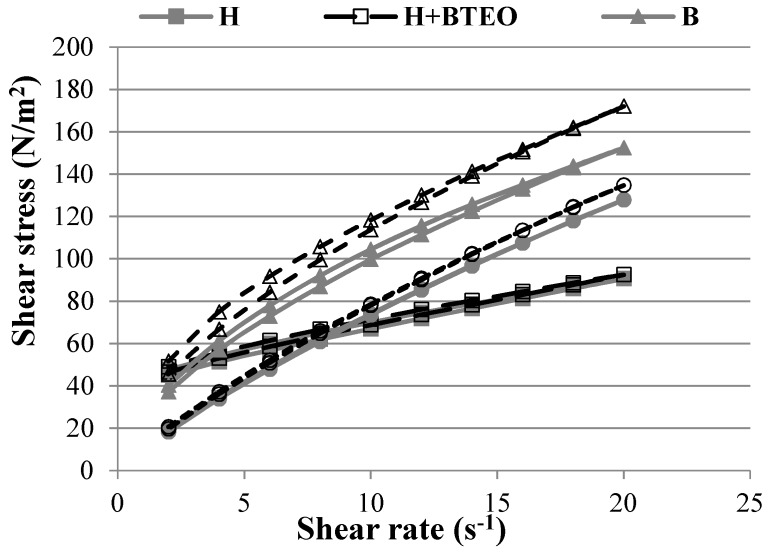
Rheograms of formulations without (H, B, O) and containing essential oil of *Bidens tripartita* (H + BTEO, B + BTEO, O + BTEO).

Formulated hydrogel and bigel possessed pseudoplastic characteristics with a shear-thinning property (Figure 2). Furthermore, these gels were characterized by thixotropy, visible as the hysteresis loops. In thixotropic systems, viscosity decreases over time at a steady shear rate. With removing the shear stress, a viscosity increment to the initial value is observed. Interestingly, a distinctive type of rheogram was observed for the oleogel (Figure 2). An almost linear dependence between shear rate and shear stress, which is typical for the Bingham model rather than pseudoplastic flow, was observed. The Bingham model describes a viscoplastic material that behaves as an elastic solid at low stresses and flows as a viscous fluid at high stress. In addition, the hysteresis loop of the oleogel indicates that its thixotropic properties are negligible. The hysteresis loops may be caused by the rigid structure of oleogel, created with using fumed silica and castor oil and resulting from a strong particle-particle interaction which lead to a firm network formation.

Local drug carriers should be characterized by optimal application properties. The textural parameters provide information about the response to an external force, and are crucial to verifying the removal facility of preparations from the container, or their spreadability on the skin surface. Firmness is the maximal force needed to accomplish a deformation, compressibility is the work needed to deform the formulation during the compression of the probe, and adhesiveness is defined as the work overbear forces between the investigated sample and the model adhesive layer. The results of the texture analysis (Table 2) confirmed findings from the rheological measurements. The highest values of the mechanical parameters were recorded in the case of the bigel containing BTEO and the lowest for the alginate hydrogel, which can be attributed to the nature of the ingredients used. Higher values for firmness, compressibility and adhesiveness observed for the bigel and oleogel were possibly due to the presence of castor oil in these formulations. Formulations without BTEO possessed slightly lower firmness, compressibility and adhesiveness.

**Figure 3:**
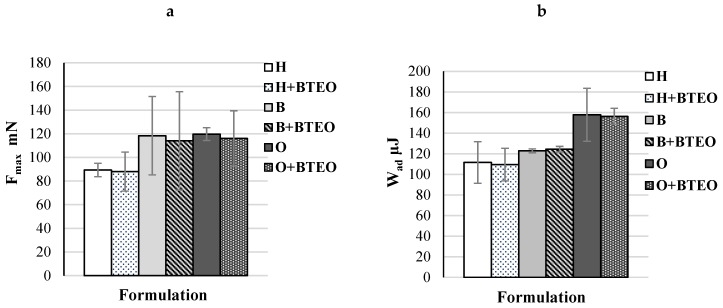
Ex vivo bioadhesive properties of formulations without (H, B, O) and containing essential oil of *Bidens tripartita* (H + BTEO, B + BTEO, O + BTEO) determined as maximum.detachment force F_max_ (**a**) and work of adhesion W_ad_ (**b**).

Bioadhesion is phenomenon when two materials are joined for a prolonged time by interfacial energies [12]. The bioadhesive properties of the vehicles used as topical drug carriers enable them to adhere to the skin, and as a result, elongate the drug retention. The results of the experiments carried out using hairless mice skin as a model of the adhesive layer are demonstrated in Figure 3. All prepared formulations were characterized by bioadhesive properties, and the greater values of F_max_ and W_ad_ were noticed in the case of the oleogel and bigel, which were characterized by higher viscosity and higher values of mechanical properties. No significant difference in the bioadhesiveness of preparations containing BTEO and placebo formulations were noted.

The antifungal activity of the hydrogel supplemented with 100 mg/g BTEO concentration was demonstrated against all tested *Candida* strains, except for *C. parapsilosis* (clinical strain). Essential oils are well-known antifungal agents. Many of them inhibited both the growth and the activity of *Candida* strains more efficiently than known antifungal drugs [13]. The highest activity was observed against *C. tropicalis* and *C. krusei* (3.7 mm difference compared to hydrogel without BTEO). In the case of the bigel formulation with essential oil, the growth inhibition zone diameters were observed in *C. glabrata* (mean of diameters 8.0 mm), *C. tropicalis*, and both *C. parapsilosis* strains (mean 7.7 mm). The minimum antifungal activity of the oleogel in combination with BTEO (mean 7 mm) was demonstrated only for the clinical *C. krusei* strain, and for individual measurements of *C*. *parapsilosis* ATCC 22019, *C. tropicalis* and *C. glabrata* (mean of diameters 6.3 mm). The results of the antifungal activity of the prepared formulations are shown in Table 3.

Among all of the tested gel formulations combined with BTEO, the hydrogel-based formulation appears to be the preferred combination that exhibited antifungal activity against all tested *Candida* strains. Interestingly, in the case of *C. parapsilosis* strains, the antifungal activity of the hydrogel itself was noted. A similar phenomenon in the antifungal activity of the carrier, oleogel, was noted in the case of *C. krusei* strains. The antifungal activity of the oleogel-based formulation was observed in the form of a zone with a reduced growth density of the tested strain. In the case of the bigel, its antifungal activity was not demonstrated against *C. krusei* strains, whereas the combination with BTEO resulted in the appearance of a growth inhibition zone up to 7.3 mm on average. Irrespective of the emerging full activity of the essential oil, the appearance of the zone in the form of the reduced growth density of the tested strain was observed. The above phenomena could possibly be related to the specificity of the particular species of *Candida*. The obtained results of antifungal activity tests demonstrate that our formulations had inhibitory effects on the growth of fungi (see Table 3). The essential oil consisted of a complex of numerous components. Possible synergistic and antagonistic effects of these phytoconstituents play an important role in *Candida species* inhibition. Previous studies on the antifungal activities of essential oils of other *Bidens* species have shown that they have varying degrees of growth inhibition effects against pathogenic fungal species [14,15,16]. 

## 3. Materials and Methods 

### 3.1. Materials and Methods

Silicon dioxide (Aerosil^®^ 200) was received as a gift sample from Evonik Industries AG, (Hanau, Germany), tocopheryl acetate was purchased from Fagron (Kraków, Poland), castor oil and glycerol 85% were from PPH Galfarm Sp. z.o.o, (Kraków, Poland), 2-bromo-2-nitropropane-1,3-diol (bronopol), Tween 80 and sodium alginate were from Sigma Aldrich, (Steinheim, Germany). Hairless mouse skin was obtained from the Experimental Medicine Center of the Medical University of Białystok. The skin was collected from Cby.Cg-Foxn1nu/cmdb hairless mice intended for the collection of organs. This procedure did not require the approval of the Local Ethical Committee for Experiments on Animals. Samples of the skin were frozen at −20°C prior to testing. After defrosting, the samples were split into 5-mm-diameter fragments. The studies were performed on normal cell line—a human dermal fibroblasts. The cell line was obtained from American Type Culture Collection (Manassas, VA, USA). 

### 3.2. Plant Material and Essential Oil Preparation

Aerial parts of *B. tripartita*, during the full blossom, were collected in the Bielsk Podlaski area (Poland) from July 28 to August 06, 2016. The identification of plant material was carried out by Michał Tomczyk. A voucher specimen of the plant (No. BT97005) has been deposited at the Department of Pharmacognosy, Medical University of Białystok (Poland). The oil (BTEO) (0.03%) was hydrodistilled (3 h) from the dried material (20 kg) using a Cleveger-type apparatus [17]. The chemical constituents of the BTEO were analyzed by the GC/MS technique.

### 3.3. GC-MS Analysis of Essential Oil

A GC-MS evaluation of the oil was conducted on a 7890A gas chromatograph and a 5975C VL mass spectrometer (Agilent, Santa Clara, CA, USA) applying the HP-5MS capillary column: 30 m × 0.25 mm, 0.25 µm film thickness. One microlitre of the sample, mixed with hexane (1:100 *v*/*v*), was injected using a split ratio (20:1) and an inlet temperature of 280 °C. The carrier gas was helium, and the flow rate was 1.0 mL min^−1^. The temperature of the column rose from 50 °C to 300 °C at a rate of 4 °C min^−1^. For GC-MS detection, an electron ionization system was used with an ionization energy of 70 eV and a mass range of 30 to 650 *m*/*z*. The composition of BTEO was determined by comparison of the relative retention indexes and the mass spectra of BTEO constituents with retention indexes indicated by the literature and mass spectra available in a data library (NIST ver. 2.0, 2012).

### 3.4. Cell Viability Assay

The cytotoxicity of BTEO on human fibroblasts was determined via an MTT test (Sigma-Aldrich, Germany) according to a modified Carmichael’s method. The 10^5^ cells were plated on 96-well plates. After reaching 90% confluency, the cells were incubated with 50, 100, 150 and 200 mg/g of the studied oil for 24 h. As a positive control, we used cells incubated with 100 μM of H_2_O_2_. Following the incubation, DMEM was removed, and the cells were washed twice with PBS. Afterward, 20 µL of the MTT solution (2 mg of MTT dissolved in 1 mL of PBS) was added to each well and incubated for 60 min. The product of the conversion - formazan derivatives was dissolved in isopropanol, after MTT solution removal. The absorbance was measured applying the microplate reader at the 570-nm wavelength (Asys UVN 340). The number of viable cells was calculated as a percentage of the control.

### 3.5. Comet Assay for Genotoxicity Testing

Human fibroblasts were used for this study. Cells were cultured on 6-well plates at 37 °C, 5% CO_2_ in DMEM (Gibco) supplemented with 10% FBS (Gibco) and penicillin/streptomycin (Gibco), for 72 h. Subsequently, the confluent cells were incubated for 24 h with test concentrations (*v*/*v*) of the oil (50, 100, 150 and 200 mg/g). As a positive control, cells incubated with 100 μM of H_2_O_2_ were used. Afterward, the medium was removed, and the cells were trypsinized, suspended in fresh medium and centrifuged. Next, 10 μL of the cell pellet was resuspended in 75 μL of a 0.5% low melting agarose solution (LMPA). Thereafter, the cell suspension was applied to the cooled primary slides coated with 1% regular agarose solution (NMA) and covered with a coverslip for approximately 5 min. After that step, the coverslip was detached, and the third LMPA agarose layer was applied. Afterwards, the cells were lysed for 60 min in lysis buffer at 4 °C (freshly made) (2.5 mol/L NaCl, 10 mmol/L Tris, pH 10; 1% sodium sarcosinate, 10% dimethylsulfoxide, 100 mmol/L EDTA, and 1% Triton X-100 (Sigma Chemical Laboratories)) without access to light and incubated for 20 min in an electrophoresis buffer (300 mmol/L NaOH, 1 mmol/L EDTA; pH 13). Electrophoresis was conducted at 300 mA and25 V. Afterwards, the slides were rinsed three times in the neutralizing buffer, stained with ethidium bromide (Sigma-Aldrich), observed with a fluorescence microscope. The obtained photos were analyzed by applying the CometAssay Lite IV software (π Perceptive Instruments, UK). Next, 50 cells were analyzed per sample point. Comet tail moment was expressed as a percent of the control value. 

### 3.6. Preparation of Gel Formulations with Essential Oil of Bidens tripartita

A hydrogel, bigel and oleogel were prepared using an RZR 2020 mechanical stirrer (Heidolph Instruments, Schabach, Germany). The composition of the designed formulations is shown in Table 4.

For the preparation of the hydrogel, gelling agent (sodium alginate, 5% *w*/*w*) was gradually added to purified water and stirred using the mechanical stirrer. Then, during continuous mixing, bronopol and glycerol were added. The essential oil of *B. tripartita* (BTEO) at a concentration of 100 mg/g was uniformly dispersed in the hydrogel. The oleogel was prepared by dissolving Tween 80 in heated castor oil (70 °C) with continuous mechanical stirring at 300 rpm, followed by dispersion of the gelling agent Aerosil^®^ 200 in the oil. After complete mixing of the gelling agent, tocopheryl acetate was added as an antioxidant. When the mixture was homogeneous, heating was stopped to gradually solidify to form the oleogel. BTEO at the same concentration as the hydrogel was uniformly dissolved in the oleogel. The bigel was produced by separately preparing the oleogel and hydrogel, and then mixing them at room temperature. The bigel formulation was made at an oleogel to hydrogel weight ratio of 30:70. During the bigel preparation, BTEO was dissolved in the oleogel, then the oleogel was mixed with an alginate hydrogel under constant mechanical stirring at 800 rpm. The concentration of BTEO in the designed preparation was 100 mg/g. A control hydrogel, bigel and oleogel without BTEO (placebo) were also prepared.

### 3.7. Quality Evaluation of Designed Gel Formulations

#### 3.7.1. pH Determination

The pH was measured using an Orion 3 Star pH-meter glass electrode (Thermo Scientific, Waltham, MA, USA). The data shown is the average of six experiments.

#### 3.7.2. Viscosity Measurement and Determination of Rheological Properties

Viscosity measurements at a shear rate of 10.00 s^−1^ were conducted at 25 ± 1 °C with a Brookfield viscometer (RVDV-III Ultra, Brookfield Engineering Laboratories, Middlebro, MA, USA) using the cone/plate CPA52Z. The data shown is the average of six experiments.

#### 3.7.3. Texture Analysis

Texture properties of the prepared formulations were examined by a TA.XT Plus Texture Analyser (Stable Micro System, Godalming, UK) using the Texture Exponent 32 software [18,19].

#### 3.7.4. *Ex vivo* Bioadhesive Properties

Bioadhesiveness was evaluated by the TA.XT.Plus Texture Analyser (Stable Micro Systems, Godalming, UK) with hairless mouse skin as an adhesive layer. The skin was stored at −20 °C for maximum four weeks. Before the experiment, the skin was defrosted and cut into 5 mm diameter pieces, then thawed in a physiological saline solution (0.9% NaCl) at 25 ± 0.5°C for 30 min. Parameters of the study were as follows: pretest speed—0.5 mm∙s^−1^, test speed—0.1 mm∙s^−1^, contact time—120 s, post-test—0.1 mm∙s^−1^, applied force—0.5 N. The adhesive properties were expressed as the maximum detachment force (F_max_) and the work of adhesion (W_ad_). W_ad_ was computed using the formula:

W_ad_ = A × 0.1 × 1000 (1)

where A—the area under the force versus distance curve, multiplication by 0.1—conversion time measurement to distance (the sampler was raised at 0.1 mm·s^−1^), then multiply by 1000 in order to express the result in units of work µJ. The results were reported as the mean of six tests [20,21].

### 3.8. Antifungal Activity of BTEO Formulations

Analysis of BTEO activity against *Candida* spp. yeast was performed using the modified agar-well diffusion method [22,23,24]. In the first step, 200 mg/mL stock solution of BTEO was prepared in DMSO. Subsequently, series of two-fold dilutions were prepared with a working concentration range of 200–1.56 mg/mL. Analysis of BTEO activity against *Candida* spp. yeast was performed on RPMI agar supplemented with MOPS (Biomaxima, Poland). The BTEO oil solutions (volume of 50 µL; oil content range 10–0.08 mg) were placed in 6 mm diameter wells that were previously cut from the inoculated plates. The RPMI plates were incubated under aerobic conditions at 35 °C for 24 h [23], and the growth inhibition zone diameters were measured in millimeters. The aim of the initial phase of the study was to determine the antifungal activity of BTEO in the above method at the lowest possible concentration. Based on the results obtained in the first phase of the study, a 100 mg/g BTEO concentration was selected for the preparation of three formulations based on a oleogel, bigel and hydrogel. In the next stage of the study, reference (*C. krusei* ATCC 6528, *C. parapsilosis* ATCC 22019) and clinical strains of *Candida* spp. were used (*C. krusei, C. parapsilosis, C. albicans, C. glabrata, C. tropicalis*). In the gel activity investigation, we utilized the agar-well diffusion method described for the initial phase of the study. Approximately 50 µg of the particular gel supplemented with BTEO was applied to the wells. The activity of BTEO at corresponding concentrations in DMSO (50 µL) was analyzed in the same RPMI plates. Antifungal activity of the gels supplemented with *B. tripartitia* essential oil was determined in three technical repetitions. Solutions of ketoconazole and clotrimazole in DMSO and commercially available products with Nizoral^®^ and Clotrimazol^®^ were used as a positive controls in antifungal susceptibility testing, respectively.

## Figures and Tables

**Table 1:** pH and viscosity of the prepared formulations without (H, B, O) and containing essential oil of *Bidens tripartita* (H + BTEO, B + BTEO, O + BTEO).

Formulations	pH	Viscosity * (mPa∙s)
H	7.1 ± 0.02	6694 ± 94
B	7.3 ± 0.00	10451 ± 57
O	6.2 ± 0.02	7402 ± 125
H + BTEO	7.0 ± 0.00	6879 ± 121
B + BTEO	7.2 ± 0.01	11833 ± 165
O + BTEO	6.4 ± 0.01	7458 ± 139

* Viscosity was tested at 10.00 s^−1^.

**Table 2:** Textural properties of the designed formulations without (H, B, O) and containing essential oil of *Bidens tripartita* (H + BTEO, B + BTEO, O + BTEO).

Formulations	Firmness (g)	Compressibility (g·s)	Adhesiveness (g·s)
H	50.7 ± 2.6	103.2 ± 9.3	−110.6 ± 9.9
B	66.6 ± 1.5	135.1 ± 1.6	−141.9 ± 0.5
O	62.2 ± 0.4	130.6 ± 1.7	−131.3 ± 0.4
H + BTEO	51.0 ± 0.8	105.8 ±1.6	−110.8 ± 0.4
B + BTEO	82.2 ± 0.8	165.8 ± 2.5	−177.4 ± 2.3
O + BTEO	63.0 ± 1.4	130.9 ± 4.0	−132.2 ± 1.4

**Table 3:** Antifungal activity of selected oleogel, bigel, and hydrogel-based formulations in combination with BTEO (100 mg/g) against *Candida* spp. yeasts.

Strains	Formulations (100 mg/g)						BTEO	N	K	DMSO
	O	O + BTEO	B	B + BTEO	H	H + BTEO				
	Mean of Three Repetition of Growth Inhibition Diameter (mm)									
*C. glabrata* 7771	6.0	6.3	6.0	8.0	6.0	8.0	8.7	29.0	19.7	6.0
*C. krusei* ATCC 6258	6.0 * (14.0)	6.0 * (13.0)	6.0	6.0 * (10.3)	6.0	9.7	11.7	29.7	26.3	6.0
*C. albicans* 7562	6.0	6.0	6.0	6.0	6.0	7.7	8.7	15.0 **	11.3 **	6.0
*C. albicans* 7389-3	6.0	6.0	6.0	6.0	6.0	8.7	8.7	23.7	15.7	6.0
*C. tropicalis* 7389-2	6.0	6.3	6.0	7.7	6.0	9.7	9.0	36.0	23.3	6.0
*C. krusei* 7525	6.0 * (13.3)	7.0 * (13.3)	6.0	7.3 * (10.0)	6.0	8.3	9.3	23.3	23.7	6.0
*C. parapsilosis* ATCC 22019	6.0	6.3	6.0	7.7	8.7	10.3	11.0	40.7	27.7	6.0
*C. parapsilosis* 7448	6.0	6.0	6.0	7.7	12.0	11.7	12.3	39.3	25.3	6.0

N—Ketoconazole, K—Clotrimazole; *—zone of reduced growth density; **—growth inhibition zone with blurred boundary.

**Table 4:** Composition of the designed semisolid preparations (hydrogel, bigel and oleogel) without (H, B, O) and containing essential oil of Bidens tripartita (H + BTEO, B + BTEO, O + BTEO).

Ingredient (g)	Formulations					
	H	H + BTEO	B (O:H Ratio 30:70)	B + BTEO (O:H Ratio 30:70)	O	O + BTEO
BTEO	-	10.0	-	10.0	-	10.0
Sodium alginate	6.5	6.5	4.55	4.55	-	-
Glycerol 85%	10.0	10.0	7.0	7.0	-	-
Bronopol	0.01	0.01	0.007	0.007	-	-
Purified water (up to)	100.0	100.0	70.0	70.0	-	-
Silicon dioxide (Aerosil^®^200)	-	-	1.2	1.2	4.0	4.0
Tween 80	-	-	0.3	0.3	1.0	1.0
Tocopheryl acetate	-	-	0.015	0.015	0.05	0.05
Castor oil (up to)	-	-	30.0	30.0	100.0	100.0

## References

[1] Pozharitskaya O.N., Shikov A.N., Makarova M.N., Kosman V.M., Faustova N.M., Tesakova S.V., Makarov V.G., Galambosi B. (2010). Anti-inflammatory activity of a HPLC-fingerprinted aqueous infusion of aerial part of *Bidens tripartita* L.. Phytomedicine.

[2] Orhan N., İçöz Ü.G., Altun L., Aslan M. (2016). Anti-hyperglycaemic and antioxidant effects of *Bidens tripartita* and quantitative analysis on its active principles. Iran. J. Basic Med. Sci..

[3] Uysal S., Ugurlu A., Zengin G., Baloglu M.C., Altunoglu Y.C., Mollica A., Custodio L., Neng N.R., Nogueira J.M.F., Mahomoodally F. (2018). Novel *in vitro* and *in silico* insights of the multi-biological activities and chemical composition of *Bidens tripartita* L.. Food Chem. Toxicol..

[4] Lupuşoru R.V., Mititelu-Tarţău L., Sandu R.B., Popa G., Zagnat M., Lupuşoru C.E. (2016). Experimental investigations on the effects of *Bidens tripartita* extracts in nociceptive reactivity. Farmacia.

[5] Wolniak M., Tomczykowa M., Tomczyk M., Gudej J., Wawer I. (2007). Antioxidant activity of extracts and flavonoids from *Bidens tripartita*. Acta Pol. Pharm..

[6] Tomczykowa M., Leszczyńska K., Tomczyk M., Tryniszewska E., Kalemba D. (2011). Composition of the essential oil of *Bidens tripartita* L. roots and its antibacterial and antifungal activities. J. Med. Food.

[7] Rehman K., Zulfakar M.H. (2014). Recent advances in gel technologies for topical and transdermal drug delivery. Drug Dev. Ind. Pharm..

[8] Singh Malik D., Mital N., Kaur G. (2016). Topical drug delivery systems: A patent review. Expert Opin. Ther. Pat..

[9] Tan X., Feldman S.R., Chang J., Balkrishnan R. (2012). Topical drug delivery systems in dermatology: A review of patient adherence issues. Expert Opin. Drug Deliv..

[10] Kaskoniene V., Kaskonas P., Maruska A., Ragazinskiene O. (2011). Chemical composition and chemometric analysis of essential oils variation of *Bidens tripartita* L. during vegetation stages. Acta Physiol. Plant..

[11] Kaskoniene V., Kaskonas P., Maruska A., Ragazinskiene O. (2013). Essential oils of *Bidens tripartita* L. collected during period of 3 years composition variation analysis. Acta Physiol. Plant..

[12] Palacio M.L., Bhushan B. (2012). Bioadhesion: A review of concepts and applications. Philos. Trans. R. Soc..

[13] Bona E., Cantamessa S., Pavan M., Novello G., Massa N., Rocchetti A., Berta G., Gamalero E. (2016). Sensitivity of *Candida albicans* to essential oils: Are they an alternative to antifungal agents?. J. Appl. Microbiol..

[14] Deba F., Xuan T.D., Yasuda M., Tawata S. (2008). Chemical composition and antioxidant, antibacterial and antifungal activities of the essential oils from *Bidens pilosa* Linn. var. radiata. Food Control.

[15] Tomczykowa M., Tomczyk M., Jakoniuk P., Tryniszewska E. (2008). Antimicrobial and antifungal activities of the extracts and essential oils of *Bidens tripartita*. Folia Histochem. Cytobiol..

[16] Rybalchenko N.P., Prykhodko V.A., Nagorna S.S., Volynets N.N., Ostapchuk A.N., Klochko V.V., Rybalchenko T.V., Avdeeva L.V. (2010). *In vitro* antifungal activity of phenylheptatriyne from *Bidens cernua* L. against yeasts. Fitoterapia.

[17] (2014). European Pharmacopoeia.

[18] Wroblewska M., Winnicka K. (2015). The effect of cationic polyamidoamine dendrimers on physicochemical characteristics of hydrogels with erythromycin. Int. J. Mol. Sci..

[19] Jin S.G., Yousaf A.M., Son M.W., Jang S.W., Kim D.W., Kim J.O., Yong C.S., Kim J.H., Choi H.G. (2015). Mechanical properties, skin permeation and in vivo evaluations of dexibuprofen-loaded emulsion gel for topical delivery. Arch. Pharm. Res..

[20] Carvalho F.C., Calixto G., Hatakeyama I.N., Luz G.M., Gremião M.P., Chorilli M. (2013). Rheological, mechanical, and bioadhesive behavior of hydrogels to optimize skin delivery systems. Drug Dev. Ind. Pharm..

[21] Sosnowska K., Szekalska M., Winnicka K. (2016). The effect of PAMAM dendrimers with amine or hydroxyl terminal groups on the bioadhesive properties of hydrogels with clotrimazole. Polimery.

[22] Winnicka K., Wroblewska M., Wieczorek P., Sacha P.T., Tryniszewska E. (2012). Hydrogel of ketoconazole and PAMAM dendrimers: Formulation and antifungal activity. Molecules.

[23] Arendrup M.C., Guinea J., Cuenca-Estrella M., Meletiadis J., Mouton J.W., Lagrou K. EUCAST DEFINITIVE DOCUMENT E.DEF 7.3. Method for the Determination of Broth Dilution Minimum Inhibitory Concentrations of Antifungal Agents for Yeasts (2015). http://www.eucast.org.

[24] Winnicka K., Wroblewska M., Wieczorek P., Sacha P.T., Tryniszewska E. (2013). The effect of PAMAM dendrimers on the antibacterial activity of antibiotics with different water solubility. Molecules.

